# Exposure of the *Plasmodium falciparum *clonally variant STEVOR proteins on the merozoite surface

**DOI:** 10.1186/1475-2875-10-58

**Published:** 2011-03-14

**Authors:** Ayman Khattab, Seppo Meri

**Affiliations:** 1 Malaria Research Laboratory, Department of Bacteriology and Immunology, Haartman Institute, University of Helsinki, Helsinki, Finland

## Abstract

**Background:**

*Plasmodium falciparum *merozoites are free invasive forms that invade host erythrocytes in iterative cycles in the presence of different arms of the immune system. Variant antigens are known to play a role in immune evasion and several gene families coding for variant antigens have been identified in *P. falciparum*. However, none of them have been reported to be expressed on the surface of merozoites.

**Methods:**

Flow cytometry, immunofluorescence microscopy, and immunoblotting assays were performed to assess surface exposure, membrane association and stage specific expression of the STEVOR family of variants proteins, respectively.

**Results:**

Using a polyclonal antibody (anti-PFL2610w) with a broad specificity towards different STEVOR variants, the STEVOR proteins were identified on the surface of non-permeabilized/non-fixed merozoites in flow cytometry assays. Anti-PFL2610w antibody showed that several STEVORs were expressed in the trophozoite stage of the parasite but only one variant was integrated into the merozoite membrane. Moreover, this antibody failed to identify STEVORs on the surface of the parent schizont infected erythrocytes (IE) although they were readily identified when schizont IE were permeabilized.

**Conclusions:**

These data suggest for a role for STEVOR in immune evasion by *P. falciparum *merozoites to allow successful invasion of erythrocytes. Additionally, the expression of STEVORs in the schizont stage may only represent a step in the biogenesis process of the merozoite surface coat.

## Background

Of the four species causing human malaria, *Plasmodium falciparum *is the most dangerous. It causes the highest rates of complications and mortality in the tropical and subtropical regions worldwide. It was estimated for the year 2002 that about 500 million people suffered from *P. falciparum*-malaria and about one million died [1]. The remarkable ability of the *P. falciparum *parasite to achieve this in comparison to other Plasmodium species is due to its capability to infect all ages of human erythrocytes leading to a higher parasite load. Additionally, the *P. falciparum *trophozoite and schizont IE stages have a unique competence to cytoadhere to a variety of human endothelia to escape from the circulation and avoid clearance by the spleen until merozoites become released and invade new erythrocytes. In malaria endemic areas, immunity develops against mild or uncomplicated *P. falciparum *malaria over a long period of time during which individuals are repeatedly infected with the parasite. On the other hand, immunity against severe forms of malaria such as severe anaemia and cerebral malaria take shorter periods to develop. A broad immunity to most forms of malaria is reached at adulthood and declines when an individual leaves the endemic area [2-4]. Despite acquiring immunity to malaria, the parasite persists and causes an asymptomatic infection in adults living in areas where malaria is endemic. Thus infected adults serve as a reservoir for the parasite in hyperendemic areas where malaria is seasonal and mosquitoes disappear during the dry seasons.

Successful merozoite invasion of new erythrocytes in the iterative growth cycle of the *P. falciparum *parasites is a prerequisite for parasite persistence in adults in endemic areas. This appears to occur despite acquiring immunity that limits both severe and mild forms of malaria. It also indicates that the parasite must have effective tools to complete the invasion steps (attachment, reorientation, engulfment and entry) without being harmed by the immune system. Proteins that possess highly variable sequences and encoded by multi-copy gene families are lead candidates for such tools. The *var*, *rif *and *stevor *are known *P. falciparum *multi-copy gene families that undergo antigenic variation [5], they could thus be involved in merozoite escape from the immune system attack.

STEVORs encoded by the *stevor *gene family are the most widely expressed variant antigens in the different stages of the *P. falciparum *parasite. They were found in the late trophozoite and schizont IE stages in flattened vesicular structures known as Maurer's clefts (MC) localized in the erythrocyte cytoplasm [6]. Further studies localized STEVORs to the IE membrane by electron microscopy [7,8] or to the IE surface by flow cytometry [9]. STEVORs were also detected in the gametocyte and sporozoite stages of the parasite [10]. The *stevor *family is represented by 39 members in the 3D7 genome [11]. *stevor *gene transcription peaks at the mid-trophozoite stage [6], 28 h post invasion (p.i.) and only a subset of stevor genes are transcribed at any given time in a population of parasites [12]. Evidence for clonal variation in *stevor *expression was reported at the transcription level [12] and at the protein level [8,9]. STEVORs share protein architecture with proteins encoded by the *rif *and the *Pfmc-2TM *families and altogether constitute what is described as the TM superfamily [13]. This architecture consists of a signal sequence, a hypervariable (HV) region surrounded by two predicted transmembrane domains and a cytoplasmic tail. The HV region is of varying length and up to 170 and 60 amino acid residues in RIFINs and STEVORs, respectively and only three residues, on average, in the Pfmc-2TM family [13]. Members of the TM superfamily have no known function so far. In recent work evidence was presented by our group for the expression of STEVORs in the merozoite stage of the *P. falciparum *parasite. Immunofluorescence and immunoelectron microscopy localized STEVORs to the surface coat of the merozoites and to the rhoptries [8]. Localization of STEVORs to the rhoptries was also shown in a different study [14]. These data suggest that the parasite might utilize STEVORs expressed in the merozoite stage to vary antigenically as a means to establish a long-lasting persistent infection together with similar or different strategies in the other stages of the parasites. Since the accessibility of the variant antigen to the surface of the cell is an important feature in mediating immune evasion, a follow-up study was conducted to verify the exposure of STEVORs on the merozoite surface.

Here, a polyclonal anti-STEVOR antibody (anti-PFL2610w) identified STEVOR(s) on the surface of merozoites in flow cytometry assays. Similar variants were identified by the same antibody also in the parent schizont IE, but not on the surface of the intact schizont IE. Western blot analyses of protein extracts from early ring, mid-trophozoite and merozoite stages of the parasite provided evidence on the over-expression of a single STEVOR variant in merozoites. These data strengthen the suggested role of STEVORs in mediating immune evasion by the merozoites to acquire a long lasting capability of invading host erythrocytes.

## Methods

### Parasite strain and culture

The *P. falciparum *strain used in this study was the 3D7 parasite line. It was cultured in O^+ ^human erythrocytes at 5% haematocrit in RPMI-1640 medium supplemented with 0.5% Albumax II (Gibco, Carlsbad, CA, USA), 200 μM hypoxanthine (Sigma, St. Louis, MO, USA) and 20 μg/mL gentamycin (Gibco).

### Anti-STEVOR antibody

**Figure 1:**
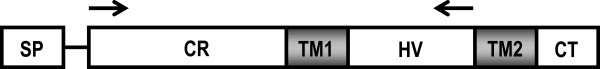
**Domain architecture of the STEVOR protein family**. The recombinantly expressed STEVOR used to produce the anti-PFL2610w antibody is delineated by the arrows. Abbreviations: signal peptide (SP), conserved region (CR), hypervariable region (HR), transmembrane domain (TM) and cytoplasmic tail (CT).

Mouse polyclonal Anti-STEVOR antibody was generated [15] against a recombinant STEVOR that included both the hypervariable and the conserved region of the PFL2610w *stevor *gene (Figure 1). Amino acid boundaries of the recombinant protein are described elsewhere [15]

### Schizont IE enrichment and isolation of free *P. falciparum *merozoites

Erythrocytes infected with *P. falciparum *ring stages were synchronized twice, 4 h apart, by sorbitol treatment (Sigma, St. Louis, MO, USA), grown to high parasitaemia (≈ 10%) of mostly schizonts and enriched to a parasitaemia of about 90 - 95% by magnet-activated cell sorting (MACS). To prepare naturally released merozoites, 44-48 hour (p.i.) schizont IE were purified by MACS and placed back into culture at 1% haematocrit in RPMI-1640 complete medium (*P. falciparum *culture medium). Every 90 min the culture was examined by microscopy for schizont IE rupture and merozoite release. When the majority of schizont IE had ruptured, free haemozoin, intact schizont IE and uninfected erythrocytes were removed by centrifugation for 4 min at 600 × g at room temperature followed by a single step of purification by passage through the MACS column. Finally, merozoites were pelleted from the flow through by centrifugation for 15 min at 2880 × g at 4°C, and washed once in PBS. Merozoite integrity was checked by Giemsa staining. Merozoites used in immunoblotting were further purified by double filtering through 3 μm and 1.2 μm Versapor membranes and pelleted by centrifugation for 15 min at 2880 × g at 4°C.

### Immunofluorescence and colocalization assays

For indirect immunofluorescence microscopy, parasites were cultured and synchronized as described above and allowed to grow for one cycle before samples were processed. IE representing the schizont stage were smeared on glass slides, air-dried and fixed for 5 min with ice-cold methanol. Smears were blocked with PBS/1% BSA for 30 min at room temperature. Slides were washed once in PBS/0.05% Tween 20 for 5 min and incubated at room temperature for 2 h with mouse anti-PFL2610w antibody (1:500) or a mix of mouse anti-PFL2610w antibody (1:500) and rabbit anti-MSP1 antibody (1:200) in PBS/1% BSA. Slides were then washed 3 × 5 min in PBS/0.05% Tween 20. Binding of the primary antibodies was visualized by Alexa 488-conjugated goat anti-mouse IgG (1:1000; Molecular Probes) when anti-PFL2610w antibody was solely used, whereas a mixture of Alexa 488-conjugated goat anti-mouse (1:1000; Molecular Probes) and Alexa 546-conjugated goat anti-rabbit (1:1000; Molecular Probes) were used when combining the analysis of both anti-PFL2610w and anti-MSP1 primary antibodies. Cell nuclei were visualized by DAPI (4',6-diamidino-2-phenylindole) (5 μg/ml; Roth, Germany). Slides were mounted on MOWIOL (Calbiochem, San Diego, CA, USA), viewed with the Olympus BX51 fluorescence microscope and images were captured using the Olympus DP70 camera with the help of DP controller software. Pre-immune sera were used to replace the anti-PFL2610w antibody for the negative control slides.

### Flow cytometry assays

Enriched schizont IE and purified free merozoites were tested for recognition of their surface by the anti-PFL2610w antibody. Schizont IE and merozoite preparations were diluted to approximately 5 × 10^6 ^cells/ml in PBS/1% BSA. Nuclei of both stages were stained with 20 μl ethidium bromide (0.1 mg/ml in PBS) per ml of cell suspension. The anti-PFL2610w antibody and the pre-immune serum were diluted to 1:400 in 100 μl of cell suspensions and incubated at 4°C for 1 hr. Schizont IE and merozoites were then washed twice in 1.5 ml PBS/1% BSA and pelleted after each wash by centrifugation at 4°C for 5 min at 800 × g and 6000 × g, respectively. Schizont IE and merozoites were resuspended in 100 μl Alexa 488-conjugated goat anti-mouse diluted to 1:500 in PBS/1% BSA and incubated at 4°C for 1 hr and then washed as above. Stained schizont IE and merozoites were resuspended in 200 μl PBS/1% BSA and analysed using the FACScan flow cytometer. Flow cytometry assays on permeabilized schizont IE were also performed. Treatment of packed schizont IE with 10 volume of 0.05% saponin in PBS for 10 min on ice renders the erythrocyte membrane freely permeable to solutes as large as soluble proteins (e.g. antibodies). Permeabilized schizont IE were washed thrice with the permeabilization buffer, pelleted after each wash by centrifugation at 3000 × g for 5 min at 4°C and treated with the same flow cytometry staining protocol as described above. Buffers used for primary and secondary antibody incubation and for washing included 0.05% saponin.

### Western blot analysis

Merozoite and saponin permeabilized early ring-IE (8-12 h) and mid-trophozoite-IE (24-30 h) stages representing a single growth cycle of the synchronized parasite were analysed by immunoblotting for the determination of STEVOR expression pattern. Trophozoite-IE and ring-IE stages were permeabilized as described above and the final pellets were boiled in 1× SDS sample buffer for 10 min. A fraction of each protein extract was loaded onto SDS-polyacrylamide gels and separated proteins were transferred to Trans-Blot transfer medium membrane (Bio-Rad, Hercules, CA, USA). The presence of erythrocyte membranes in each protein extract preparation was monitored with anti-glycophorin A antibody (abcam, USA). The membranes were probed with either anti-PFL2610w antibody or the pre-immune serum. Reactivity was visualized by goat anti-mouse IgG coupled to horseradish peroxidase (1:30 000; Pierce, Rockford, IL, USA). Blots were developed by the enhanced chemiluminescent (ECL Western blot analysis system)-based detection according to the manufacturer's instructions (GE Healthcare, Life Sciences, Bucks, UK) followed by exposure to Super RX films (Fujifilm). Blots were imaged and bands were quantified by densitometric analysis of band intensity using Kodak 1 D v. 3.5.3 software (Kodak Scientific Imaging Systems).

## Results

### Infected erythrocytes permeabilization is required for STEVOR recognition by the anti-PFL2610w antibody in flow cytometry assays

**Figure 2:**
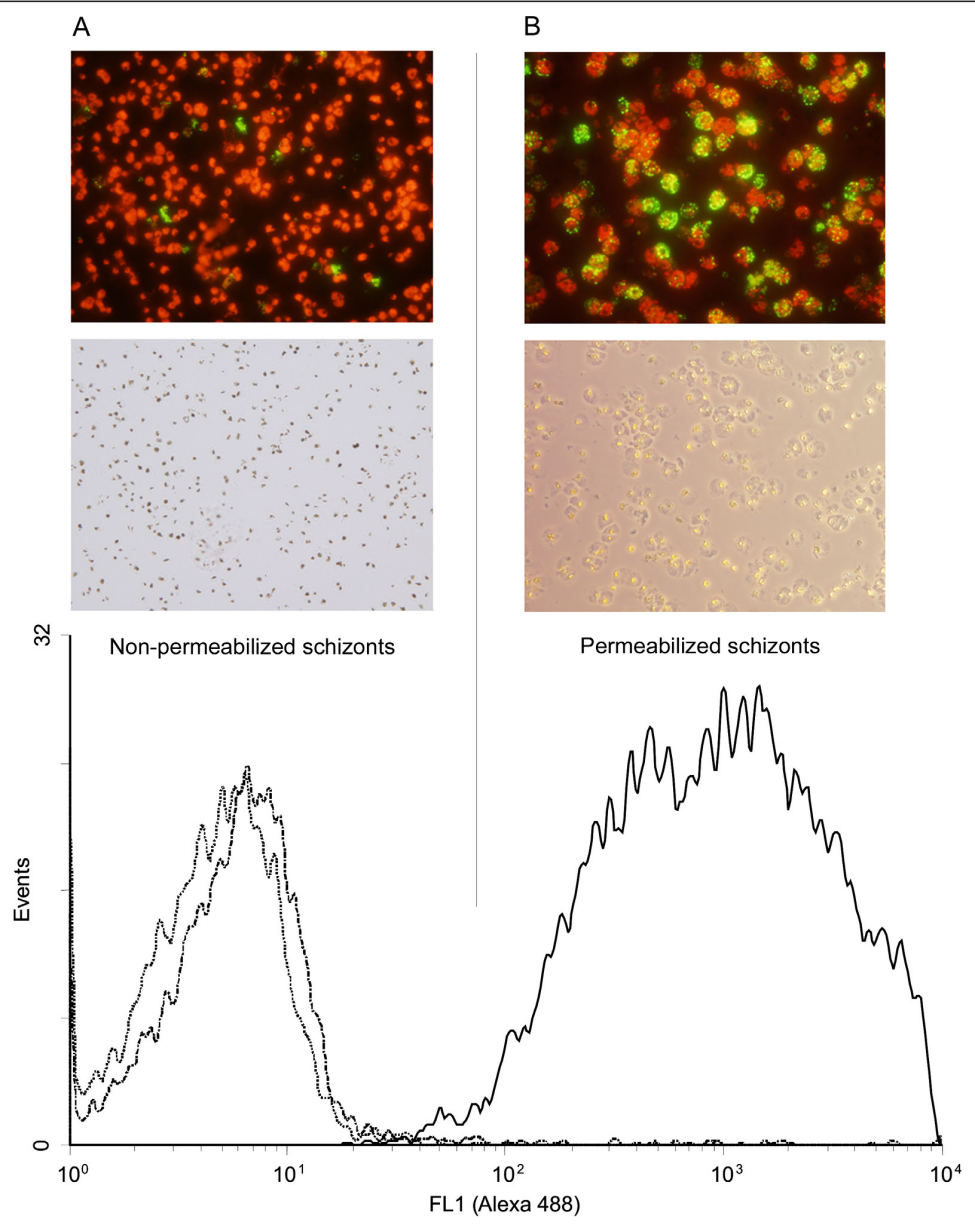
**Recognition of STEVORs in non-permeabilized and saponin-permeabilized schizont infected erythrocytes (IE)**. (A, lower panel) Flow cytometry histogram of non-permeabilized schizont IE recognized by the pre-immune serum (dot line) and by the anti-PFL2610w antibody (dot-dash line). (A, upper panel) Fluorescence microscopy image of the schizont IE that were recognized by anti-PFL2610w antibody in flow cytometry. (B, lower panel) Flow cytometry histogram of permeabilized schizont IE recognized by the anti-PFL2610w antibody (solid line). (B, upper panel) fluorescence microscopy image of the permeabilized schizont IE recognized by anti-PFL2610w antibody in flow cytometry.

The predicted secondary structure characteristics of STEVORs suggest that STEVORs' hypervariable region could acquire cell surface location and be exposed to the immune system at some stages during infection. Thus, STEVORs are speculated to be involved in antigenic variation and host parasite interaction, biological functions that are well described for the IE surface protein, PfEMP1. This is known to mediate cytoadhesion and immune evasion in *P. falciparum *infection. Earlier reports showed expression of STEVORs in the IE as well as other stages of the parasite. More recent studies have shown the expression of STEVORs at the apical end of the merozoites in immunofluorescence microscopy and on the surface of IE using flow cytometry assays and STEVOR variant-specific antibodies [9,14]. In our parallel effort to link the predicted protein organization of STEVORs with the biology and pathogenesis of the parasite, STEVORs were shown to be associated with the surface of merozoites [8]. STEVORs were also detected as released proteins from the merozoites during erythrocyte invasion [8]. These findings prompted us to ask whether the variant specific antibody that was used to localize STEVORs at the merozoite membrane can identify STEVORs on the surface of the parent schizont IE or a biologically relevant stage-specific differential expression is taking place. Thus, enriched non-permeabilized/non-fixed 3D7 schizont IE were analysed in flow cytometry assays using the antibody that we have produced against a specific STEVOR variant (PFL2610w). This antibody is capable of recognizing STEVORs associated with the merozoite membrane. Minimal binding of anti-PFL2610w antibody to the surface of schizont IE was observed as indicated by the slight shift in the relative fluorescence intensity compared to the basal level binding of the pre-immune serum antibodies (Figure 2A, lower panel). This was confirmed by immunofluorescence microscopy on the same preparation (Figure 2A, upper panel). Additionally, the immunofluorescence microscopy showed some green fluorescent staining that did not overlay well with the nuclear staining. This probably represented a population of ruptured cells. This matched the dot blot quadrant statistics showing an increase in the ethidium bromide/Alexa-488 double stained population from 2.17% with pre-immune serum to 3.86% with the anti-PFL2610w antibody (dot plot figure not shown). This observation prompted us to ask whether the anti-PFL2610w antibody could recognize intact cells if they are permeabilized, a condition close to cell rupture. Consequently, schizont IE were permeabilized by 0.05% saponin and the staining protocol was repeated as before but with the exception that 0.05% saponin was present in all solutions. Staining protocol was also performed on non-permeabilized schizont IE to control for possible generation of specific variations in the expressed STEVORs. Interestingly, flow cytometry analysis of this preparation showed a striking shift in the relative fluorescence intensity compared to the pre-immune serum (Figure 2B, lower panel). The same preparation was also analysed by immunofluorescence microscopy and schizont IE staining with anti-PFL2610w was confirmed (Figure 2B, upper panel). On the other hand, the anti-PFL2610w antibody still did not bind to the non-permeabilized schizont IE. In conclusion, these findings indicated that the anti-PFL2610w can only recognize STEVOR variants that are associated with internal schizont IE structures but not those presumably expressed on the surface.

### STEVOR is expressed on the surface of merozoites

**Figure 3:**
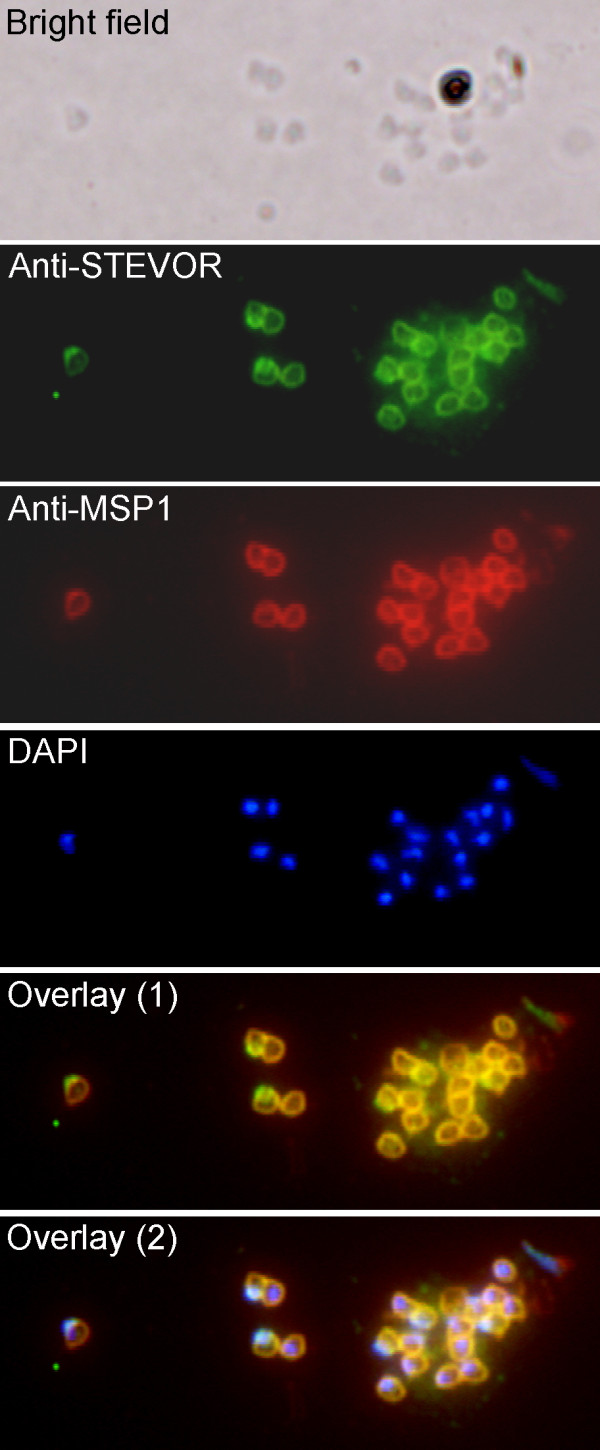
**Colocalization of STEVORs with MSP-1 at the free merozoite membranes**. Fluorescence staining using anti-PFL2610w and anti-MSP-1 antibodies was analysed in free merozoites. (A) Bright field, (B) Alexa 488 stained STEVORs, (C) Alexa 594 stained MSP-1, (D) DAPI stained parasite nuclei, (E) the overlay of STEVORs and MSP-1 (overlay 1) and (F) the overlay of STEVORs, MSP-1 and nuclei (overlay 2) images are shown.

**Figure 4:**
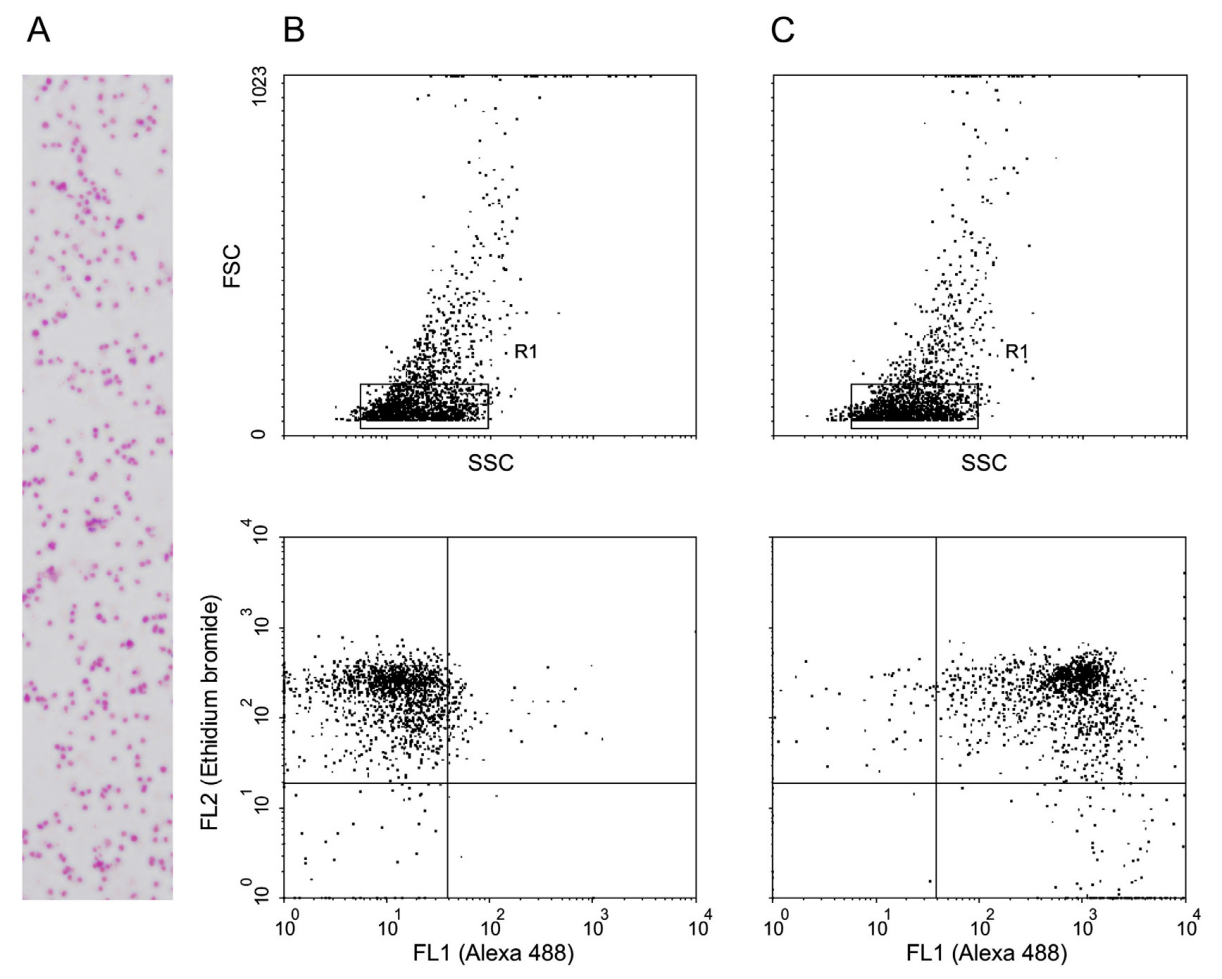
**Detection of STEVORs on the surface of merozoites by flow cytometry**. (A) Merozoite preparation stained with Giemsa. (B and C, upper panels) Dot-plots of forward scatter (FSC) to side scatter (SSC), merozoite populations are surrounded by the R1 gate. (B and C, lower panel) Dot-plots of Ethidium bromide- to Alexa 488-derived fluorescence of the gated populations. (C, lower panel) double-positive merozoite population demonstrating surface exposure of STEVORs at the upper right quadrant.

Next we wanted to study whether anti-PFL2610w antibody can recognize STEVORs in merozoites released from schizont IE that were used for the flow cytometry assay and did not show any surface labelling. A fraction of this schizont IE preparation was allowed to naturally rupture and release merozoites. Subsequently, binding of anti-PFL2610w antibody to merozoite membranes was assessed by immunofluorescence microscopy using methanol fixed merozoite slides. Remarkably, anti-PFL2610w antibody that failed to recognize the surface of non-permeabilized schizont IE in flow assays readily recognized the membranes of fixed merozoites in immunofluorescence microscopy (Figure 3). Moreover, an antibody to the well described merozoite surface protein (MSP-1) showed that both proteins occupy the same location (Figure 3). Additionally, recognition of STEVOR variant(s) in the merozoite membrane by this antibody clearly showed the association of STEVORs with the merozoite surface membrane. However, it remained unclear whether STEVORs were exposed on the surface or not, a location suspected for an antigen with such domain organization like STEVOR. To further clarify these points freshly isolated non-permeabilized/non-fixed merozoites (purity of the preparation is shown in Figure 4A) were probed with anti-PFL2610w antibody and the bound antibodies were visualized by anti-mouse Alexa-488 conjugated antibody. Interestingly, anti-PFL2610w antibody bound to the surface of merozoites represented as the Ethidium bromide/Alexa 488 double stained population (Figure 4C, lower panel). Antibodies in the pre-immune serum did not show any significant binding to the merozoite surface (Figure 4B, lower panel) supporting the specificity of the anti-PFL2610w binding. Altogether, these data showed that anti-PFL2610w could not recognize STEVOR on the surface of the schizont IE, but rather in internal structures. Moreover, STEVORs were readily indentified on the merozoites surface upon their release from the schizont IE.

### Over-expression of a single STEVOR in merozoites

**Figure 5:**
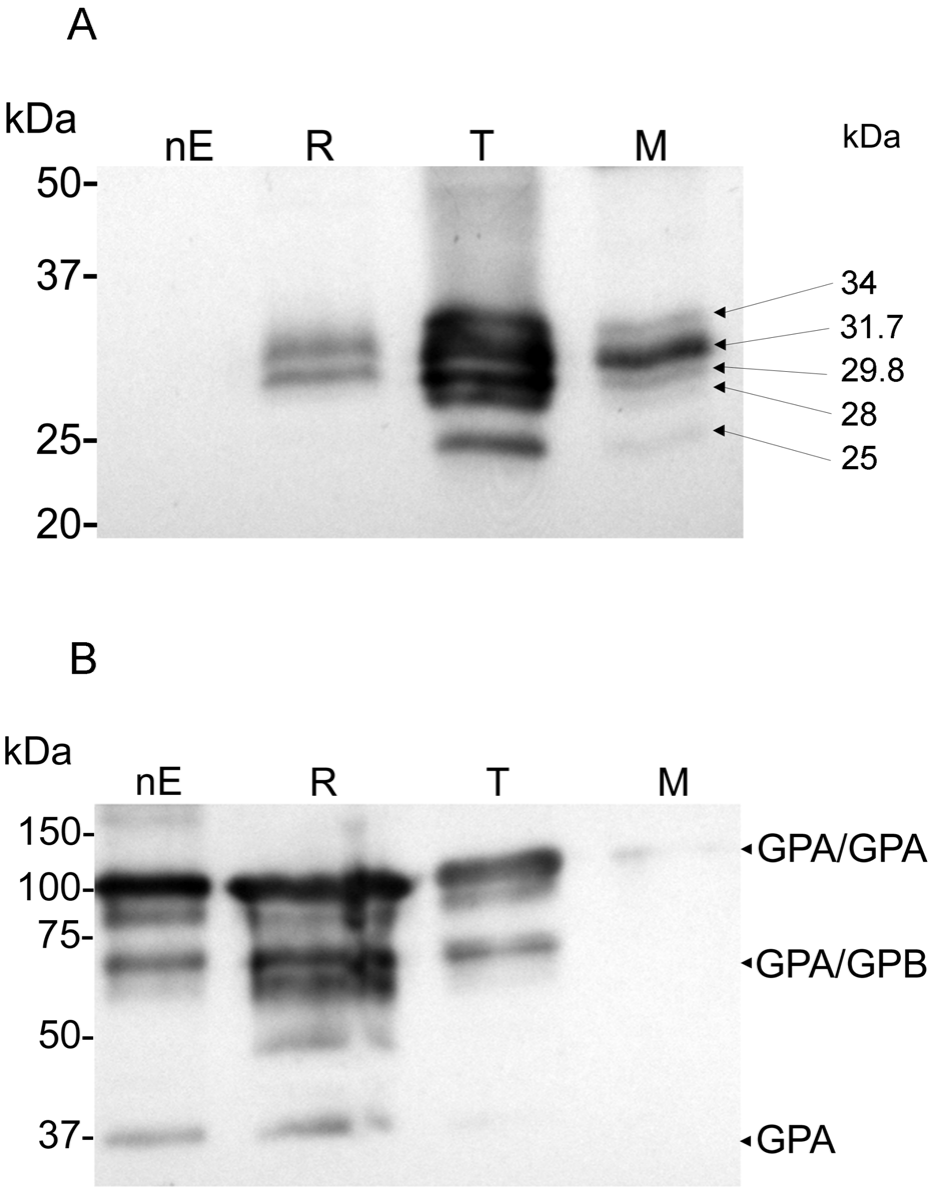
**Stage-specific expression of STEVORs**. (A) Western blot analysis of total protein from saponin permeabilized erythrocytes (nE), ring IE (R), trophozoite IE (T), and merozoites (M) probed with anti-PFL2610w. (B) A blot similar to (A) probed with anti-Glycophorin A antibody to monitor for the presence of erythrocyte membranes. Migration of molecular mass markers is indicated on the left. Estimations of the molecular masses of the identified STEVORs are indicated on the right. Abbreviations: Glycophorin A (GPA), Glycophorin B (GPB).

Anti-PFL2610w was generated against a recombinant protein that included both the variable variant specific sequence and the semi-conserved STEVOR family specific sequence (Figure 1). This antibody was successfully used to study clonal variation of STEVOR expression in NF54-derived lines in relation to their binding capacity to chondroitin sulphate A and CD36 receptors and subsequent loss of these phenotypes [8]. Therein, several STEVOR variants were identified at the late trophozoite stage by the anti-PFL2610w antibody in Western blot analyses. Moreover, the antibody identified STEVOR expression in the ring, trophozoite, schizont and merozoite stage parasites in immunofluorescence microscopy. The former data and the new finding on STEVOR expression on the surface of merozoites prompted us to ask whether a differential expression of STEVOR variants take place in these parasite stages. Therefore, SDS-soluble proteins of the early ring (8-12 h), mid-trophozoite (24-30) and merozoite stages corresponding to a single generation were analysed in a Western blot analysis using the anti-PFL2610w anti-STEVOR antibody. A total of five STEVOR variants with variable band intensities were identified by the antibody (Figure 5A and Table 1) in the stages examined. Only two variants (31.7 and 29.8 kDa) with relative band intensities of 0.56 and 0.44 respectively were identified by the anti-PFL2610w antibody in the early ring stage parasite (Figure 5A and Table 1). The transition from ring to trophozoite stage was accompanied by the expression of three more variants (34, 28 and 25 kDa) in addition to the two ring stage variants (31.7 and 29.8 kDa). These two variants retained relatively higher expression levels compared to the other variants according to the relative band intensities estimation (Figure 5A and Table 1). Interestingly, the development of mid-trophozoites into schizonts for merozoite release resulted in over-expression of a single variant (31.7 kDa) in the free merozoites (Figure 5A and Table 1). This merozoite specific variant (31.7 kDa) was one of the five variants that seemed to have nearly equal expression levels in the mid-trophozoite stage. Possible scenarios for these differences in the levels of expression between different stages include stage specific upregulation of gene expression or compartment specific localization of the expressed proteins. The latter scenario implies selective integration of specific STEVOR variants into merozoite membranes during their biogenesis. To guarantee that the merozoite preparation was devoid of IE membranes, merozoites were purified by double filtering through 3 μm and 1.2 μm Versapor membranes. This ensured that IE membranes from ruptured schizont IE were not carried over to the merozoite preparation. This was verified by detecting the glycophorin A (GPA) content in the Western blot (Figure 5B) using sample volumes identical to those used for detecting STEVOR variants. Anti-GPA detected only a minor quantity of GPA dimmer in the merozoite preparation with a band intensity value of 31.5 compared to 209, 201 and 166 for uninfected E, ring IE and trophozoite IE respectively (Figure 5B). Monomeric GPA and GPA/Glycophorin B (GPB) complex were undetectable in the merozoite preparation (Figure 5B). In conclusion, a single STEVOR variant appeared to be over represented in the merozoite stage from in vitro culture of the 3D7 *P. falciparum *parasite.

**Table 1:** Quantification of STEVOR bands identified by the anti-PFL2610w in Western blot analysis

*STEVOR variant (kDa)*	*Ring infected erythrocytes*		*Trophozoite infected erythrocytes*		*Merozoites*	
	
	Band intensity^1^	Relative intensity^2^	Band intensity	Relative intensity	Band intensity	Relative intensity
34			122	0.15	62	0.09
31.7	95.20	0.56	159	0.25	181	0.65
29.8	93.60	0.44	177	0.23	72	0.19
28			155	0.18	20	0.04
25			162	0.19	17	0.03

^1^The intensity value at the band peak.

^2^The percent intensity contribution of a band within a lane.

## Discussion

Parasitic protozoa are unicellular eukaryotic pathogens that reside in cells and/or in the extracellular fluids of their hosts. Their success as parasite pathogens depends on a series of complicated and highly evolved host adaptations that enable them to avoid destruction by the immune system. Adoption of an intracellular life cycle is perhaps the simplest solution for evading antibody responses. Another major strategy that protects both intracellular and extracellular protozoan pathogens from immune recognition is antigenic variation of proteins expressed on the surface of the pathogen. Plasmodium parasites are among those protozoa that utilize both strategies. On one hand, they acquired both an intracellular and extracellular life style and, on the other hand, they display variant antigens on the surface of some parasite stages. Antigenic diversity on the surface of *P. falciparum *IE is well represented by PfEMP1 that undergoes antigenic variation while maintaining ability to cytoadhere. There is compelling data on the exposure of this molecule on the surface of the infected erythrocyte [16,17]. Other antigens also classified as *P. falciparum *variant antigens are encoded by the multi-copy *rif *and *stevor *gene families. Analogous to what has been described for PfEMP1, original studies have reported the expression of RIFINs, encoded by the *rif *gene family, in internal structures and on the surface of the late blood stages of the parasite by the surface iodination technique [18]. While a recent report described the expression of RIFINs in a cap-like structure at the apical end of the merozoite [19], additional evidence to support the previously described surface expression of RIFINs is still lacking. STEVORs comprising another putative variant antigen family are expressed in the trophozoite, schizont, merozoite, gametocyte and sporozoite stages. They are thought to be also displayed on the cell surface; a location that would justify their amplification into a hypervariable multigenic family. A recent study showed the expression of STEVOR on the surface of late schizont using antibodies against specific variant (PF10_0395 and PFF0850c) in flow cytometry assays [9]. In a previous study we also showed the association of STEVOR with schizont IE surface by the IEM technique [8].

In the current work, STEVORs were shown to be expressed on the surface of the merozoite stage. This is the first time that a variant antigen has been shown to be expressed on the surface of extracellular *P. falciparum *stage. Unexpectedly, the current study failed to detect STEVORs on the surface of schizont IE using the anti-PFL2610w antibody which localized STEVORs to the surface of the merozoites. However, this antibody recognized STEVORs in internal structures after permeabilization of the schizont IE. These findings could simply indicate that the STEVOR variants recognized by anti-PFL2610w antibody were not exposed on the surface of the schizont IE. Instead, other variants, if any, could be exposed. In a previous attempt to link *stevor *gene sequence diversity with surface expression STEVORs were detected on the surface of schizont IE using antibodies that were made against the conserved region only [9]. It was suggested by the latter study that the predicted transmembrane domain 1 is not inserted into the erythrocyte membrane. It was also suggested that the conserved region and the variable loop are exposed on the surface through anchoring of STEVOR as a single pass membrane protein. If that were the case, the anti-PFL2610w antibody should have recognized the surface of the schizont IE as it was generated against both the conserved and the variable regions. One possible explanation for this conflict in reagent specificities relates to differences between the IgG fractions in the polyclonal antibodies that target the non-conserved epitopes on the conserved region of STEVORs. These IgG fractions should be also variant specific and their targets could be more exposed on the protein and lead to limited cross-reactivity. It is also possible that STEVORs might not be exposed at all on the IE surface. This argument should remain valid until further evidence on surface expression is presented. Moreover, the noticeable expression of STEVORs in the schizont stage may only represent a step in the biogenesis process of the merozoite surface coat. Thus, the presence of STEVORs in the schizont stage does not mean that they should be exposed on the surface because they may not function at this stage. This resembles the appearance of MSP-1 at the schizont periphery at 34.5 - 36 h (p.i) in the biogenesis process of the merozoite surface coat [20] that will ultimately lead to exposure of MSP-1 on the merozoite surface, but not on the surface of the schizont IE.

Stage-specific expression of STEVOR variants was assessed by estimating the number and the level of expression of variants identified by the anti-PFL2610w antibody in the ring, trophozoite and merozoite stages. The study revealed an over-expression of a specific variant (31.7 kDa) in the merozoites. This finding agrees with the postulation that the widespread expression of STEVOR in different stages is for serving different functions and indeed such a role should be played by stage-specific variants. RIFINs represented an example on stage-specific expression of distinct variants based on categorization of the encoding sequences and the corresponding protein localization [21]. In this report, RIFINs were grouped into two types, the A-type RIFIN that was found to be associated with the membrane of the IE or the apical region of the merozoites and the B-type RIFIN that was retained inside the parasite. Stage-specific expression of STEVOR variants based on differences in gene or upstream sequences has not been reported so far. In a transcription study *stevor *transcripts in the 3D7 trophozoites and gametocytes were similar except for a single *stevor *gene that was down regulated in gametocytes [22]. These findings suggest that some variants might only be expressed in certain stages to mediate a stage-specific function.

Moreover, an unexpected finding in the stage-specific expression of STEVORs was the detection of STEVORs (31.7 and 29.8 kDa) in the ring stage of the parasite. This is in contrast to the known first appearance of *stevor *transcripts at 22 h (p.i). It is likely that STEVORs located on the merozoite surface could have been carried into the erythrocytes during invasion and might have persisted for some time. Similar finding was also observed for the C-terminal 19-kDa fragment of the MSP-1 [20,23].

In conclusion, it was shown here that STEVORs are expressed on the surface of merozoites. This finding is in concordance with previous studies [8,14] describing STEVORs expression in the merozoite stage of the *P. falciparum *parasite. Exposure of STEVORs on the merozoite surface may contribute to immune escape of merozoites for successful erythrocyte invasion.

## Competing interests

The authors declare that they have no competing interests.

## Authors' contributions

AK and SM conceived and designed the experiments. AK performed the experiments, analysed the data, and wrote the paper. SM participated in the revision of the paper. All authors read and approved the final manuscript.
